# A comparative study of diet choice and feeding management among UK pet and child caregivers

**DOI:** 10.3389/fvets.2026.1883885

**Published:** 2026-10-12

**Authors:** Rachel H. Lumbis, Philippa S. Yam, John J. Reilly, Samantha J. Fontaine

**Affiliations:** 1 School of Biodiversity, One Health and Veterinary Medicine, College of Medical, Veterinary and Life Sciences, University of Glasgow, Glasgow, United Kingdom; 2 Department of Psychological Sciences and Health, Physical Activity and Health Group, University of Strathclyde, Glasgow, United Kingdom

**Keywords:** animal nutrition, child nutrition, companion animal, dietary pattern, family caregivers, feeding behavior, human animal interaction (HAI), one health

## Abstract

**Introduction:**

Caregivers play a central role in shaping the dietary habits and nutritional health of dependents. However, the diversity of available diets and feeding advice can complicate everyday food decisions. This qualitative study explored how caregivers make feeding decisions for their children and pets, focusing on factors influencing diet choice, feeding practices, perceptions of nutrition, and emotional attachment to dependents.

**Methods:**

UK-based caregivers aged 18 years or older, responsible for at least one child aged up to 10 years and a cat and/or dog, were recruited between July and November 2024. Semi-structured online interviews were conducted with 17 participants. Audio recordings were transcribed and analyzed thematically using an interpretive approach. Ethical approval was granted.

**Results:**

Four key themes emerged. First, participants framed nutrition as a moral responsibility across both children and pets, positioning feeding as central to health and wellbeing and shaped by knowledge, identity, emotional attachment, habits, and practical realities. Second, diet choice and feeding practices were shaped by psychosocial determinants, with caregivers’ knowledge, beliefs, routines, emotional motivations, and social norms influencing feeding decisions across both children and pets. Third, feeding dynamics differed between contexts. Children’s diets involved negotiation and bidirectional relationships shaped by preferences, routines, and agency, whereas pet feeding was more caregiver-directed, routine-based, and influenced by trust in nutritional adequacy and pet-specific needs. Fourth, feeding practices reflected relational attachment and emotional bonds.

**Discussion:**

Although pets were considered valued family members, their feeding needs were generally prioritized below those of children. Across both contexts, caregivers relied on observable cues, emotional responses, and socially shared norms to assess appropriate feeding. Caregiving beliefs and feeding philosophies transfer across contexts in complex ways, highlighting opportunities for family-focused interventions that support evidence-based nutrition for both people and animals.

## 1 Introduction

Caregivers increasingly assume active roles in making healthcare decisions for themselves and family members (1), the latter of which commonly extends to companion animals (2–5). This reflects the changing status of pets within households, with many caregivers humanizing their pets to some degree (6, 7), with some considering them children or ‘fur babies’ (2, 8–13). Attachment, defined as the ‘degree of affection that may exist between individuals and their companion animals’ (14) may contribute to this reconceptualization. Unlike children, who become more autonomous as they age, companion animals remain dependent on their caregivers, potentially reinforcing caregivers’ identity as a surrogate parent (15) or ‘pet parent’ (3). This familial positioning is reflected in caregivers’ willingness to prioritize pets’ needs, with some valuing them equal to, or above, human lives (16), favoring quality over cost when purchasing pet products (17), and sacrificing personal expenditure for their children and pets (18). These close caregiving relationships may also influence how caregivers approach decisions concerning their pets’ health and nutrition.

The pet food industry is a rapidly growing and important sector of the pet care market (19). From 2014 to 2024 the value of the UK pet food market increased over 57% from £2.6 billion (20) to £4.1 billion (18). Emotion strongly influences dog caregivers’ purchasing decisions, which food manufacturers often target through branding slogans (17, 21). Providing food and treats offers caregivers a sense of doing something good for their pet and strengthens the human-animal bond (6, 22), with dog caregivers reportedly more sensitive to the cost of human food than dog food (23). In contrast to the active influence that children and infants can have on their caregivers’ spending behavior (24, 25), pets cannot vocalize their preferences, and the level of human-animal interaction is subject to change (26). Thus, the purchase of non-essential pet products, such as grooming equipment, toys and furniture, is directly influenced by human-pet relationship (27), with greater attachment linked to increased likelihood of purchasing luxury products (28–30).

Optimal nutrition is fundamental to the health of both humans and companion animals, supporting wellbeing, longevity, disease prevention, and disease management. It is a key contributory factor to growth (31–33) early neurodevelopment (34) and life-time health outcomes (35) with influence over permanent and non-permanent morphological, physiological and behavioral changes (35–37). In children, this critical period of growth and development is also influenced by early feeding practices, dietary exposures and parental modeling, which can shape future health outcomes, eating behaviors (38–40), food consumption (41–43), dietary preferences (44–46) diet quality (34) and appetitive traits (47).

As young children and companion animals are dependent on caregivers to meet their nutritional needs, caregivers play a central role in shaping and regulating the energy intake (48) food intake, feeding environment, dietary habits and eating experience (49, 50) of dependents who cannot make informed choices for themselves. Within most households, a dietary gatekeeper assumes primary responsibility for acquiring and preparing food for the family (51). Individuals fulfilling this role exert substantial influence over dietary intake and eating habits (52), with potentially beneficial and detrimental consequences (31).

The negative impact of suboptimal diet on health is reported in children (53–55) and pets (56–59). In children, inadequate diet quality and variation contribute to increased risk of nutrient deficiencies and greater risk of obesity and metabolic dysfunction. Comparable effects are observed in pets, where unbalanced and energy-dense feeding practices contribute to dietary deficiencies and excesses and increased risk of obesity. Anthropomorphic caregivers are more likely to apply their own or their children’s preferences when feeding their pet (60, 61), yet dogs and cats have many unique distinctions. In the UK, diet is one of the leading causes of avoidable ill-health in people, with unhealthy diets accounting for 13% of all UK-based deaths (62). Recent findings from the National Diet and Nutrition Survey (NDNS) 2019–2023 show that most UK adults and children do not meet several key dietary recommendations (63). Concerning trends have been reported in the consumption of sugar, processed meat, saturated fat, salt and micronutrients (31, 63). Well-meaning, yet misguided feeding practice can have significant consequences for the health and well-being of dependents, whether children or companion animals. Appropriate dietary choices are therefore integral to effective care provision with an ultimate goal to feed for ideal health, performance and longevity.

Caregivers’ dietary beliefs, motivations and preferences can influence the health and wellbeing of their dependents, irrespective of species. Feeding is a dynamic relationship involving interactions between caregivers and dependents during food selection, consumption, and regulation (64). In child feeding, the Division of Responsibility (DOR) framework (65, 66) describes complementary roles, whereby caregivers determine what, how, where, and when food is offered, while children decide what and how much, or even whether, to eat (67, 68). This relationship can be unidirectional (69–73) reciprocal or bidirectional (74–79) and can be complex and ambiguous (74), due to the dynamic and complex nature of the parent–child relationship (47, 49). Dietary-related decisions may be deliberate or habitual (80) and are shaped by interacting individual, social and environmental factors, including personal history, biology, previous experience with food (81), values and lifestyle (82), economic circumstances and the environment (83) as well as culture, tradition and social norms (47, 61). Sense of identity also plays a key role (82, 84), but consumers often prioritize habit and convenience over personal beliefs (80).

The wide availability of foods and commercial diets can make feeding decisions challenging for both child and pet caregivers (79, 85–87). In companion animal feeding, growing skepticism toward conventional, commercially-produced pet food (88) has contributed to increased interest in non-traditional diets, such as raw, homemade, and grain-free, despite their links to health risks and nutritional deficiencies (57, 89–93). However, research has largely examined child and pet feeding in isolation, with no research directly comparing caregivers’ dietary decision-making across both contexts. Consequently, little is known about how caregivers responsible for both children and companion animals navigate feeding decisions across these caregiving contexts, or indeed whether similar psychosocial processes underpin dietary decision-making across species. In particular, the influence of cognitive and emotional factors, relational dynamics, and practical constraints has not been examined comparatively within the same caregiving context.

There is no single, internationally agreed definition of One Health, however it is commonly described as an integrated approach recognizing the interconnectedness of human, animal and environmental health (94, 95). The historical foundations of One Health can be traced to the 19th century, when Rudolf Virchow recognized the interconnectedness of human and animal health and coined the term “zoonosis” (96, 97). The link between humans, animals and public health was later recognized through the concept of “One Medicine” (98, 99) then “One World One Health” (100) and eventually “One Health” (101). While earlier conceptualizations focused primarily on health outcomes arising from human–animal interactions, contemporary One Health perspectives increasingly encompass the wider social, environmental and ecological systems in which humans and animals live (102). The current study operationalized One Health, using it as conceptual framing for the comparison of caregivers’ feeding practices and diet-related decision-making across children and companion animals within the same household. This research examined how caregivers made dietary and feeding decisions for children and pets within shared social, environmental, and household contexts, while recognizing species-specific differences in nutritional requirements, developmental stages, and dependency. By examining common influences such as caregivers’ beliefs, values, access to nutritional information, and household routines, the study applied a One Health perspective without implying equivalence between species.

This manuscript reports the direct comparison of caregivers’ self-reported factors influencing diet choice, strategies used to manage food intake, perceptions of the importance of nutrition, and attachment to their children, dogs, and cats. Study findings relating to caregivers’ claims and motivations for the feeding and avoidance of minimally processed and ultra-processed food will be reported in a subsequent publication. This study was conducted in the UK and should be considered within a Western cultural context, where companion animals are commonly regarded as family members, and caregivers may assume responsibility for the health and wellbeing of both children and pets. Feeding practice was defined as the specific strategies, actions, and goal-directed behaviors used by caregivers to influence their children’s and pets’ food intake (33, 103). Using an interpretive qualitative approach, the study provides insight into how caregivers navigate shared and species-specific influences within the household. This comparative perspective contributes to understanding the relational and contextual factors shaping feeding practices and provides a basis for considering how veterinary and medical professionals might better tailor nutrition communication and guidance to caregivers’ existing beliefs, routines and motivations.

## 2 Materials and methods

### 2.1 Ethics statement

The study was approved by the University of Glasgow MVLS College Ethics Committee (200230318). Recruitment of participants took place between 15 July 2024 and 14 November 2024. Study participants gave their full and informed consent for their de-identified data to be analyzed and included for the purposes of this research project.

### 2.2 Positionality

Qualitative research is relational, subjective, and interpretive (104, 105) and thus researcher positionality directly influences the research process and the interpretation of findings and should therefore be acknowledged (106). The lead author (R. L.) is a non-practicing Registered Veterinary Nurse with the Royal College of Veterinary Surgeons and has over two decades of experience teaching, publishing, and researching small animal nutrition. The senior author (S. F.) is also a non-practicing Registered Veterinary Nurse. Both are parents of young children and former pet caregivers, providing experiential insight into participants’ perspectives on feeding management and dietary decision-making. These authors value evidence-informed nutrition and responsive feeding practices for both children and companion animals. Ongoing reflexive discussions were held throughout data analysis to acknowledge how these perspectives could influence the interpretation of participants’ accounts, critically examine underlying assumptions, and consider alternative interpretations.

Study participants were required to be aged 18 or over, be resident in the UK and have feeding responsibility for one or more cats and/or dogs and at least one child aged up to 10 years.

### 2.3 Questionnaire design

A screening questionnaire (Supplementary File 1) was developed by R. L. and used to obtain fundamental information and to ensure that respondents were pet and child caregivers. This was developed and distributed using Qualtrics survey software (Qualtrics, Provo, UT) and shared through social media (i.e., relevant pet-centric Facebook groups) and professional networks (i.e., LinkedIn) using the snowball sampling method. Additionally, it was distributed online and in-person via the University of Glasgow’s Small Animal Hospital. The questionnaire contained 13 questions and was divided into three sections. The first section collected information about respondents’ age, settlement, education, employment, and dietary preferences. The second and third sections collected information about the number of children and pets, respectively, for which respondents had responsibility for feeding and their adopted dietary preferences. Collected information was transferred by R. L. into a spreadsheet (Microsoft® Excel® for Microsoft Office 2024, Version 17.0).

### 2.4 Sampling and recruitment

The target population was UK-based caregivers of at least one child aged up to ten years and a dog and/or cat. Participants qualified as potential interview participants if they completed the screening questionnaire in full and met all inclusion criteria. A total of 81 individuals completed the questionnaire, of which 65 qualified for inclusion as potential interview participants.

Questionnaire respondents were selected to participate in an interview using purposive sampling. This ensured the inclusion of participants who could provide diverse and contextually relevant insights according to shared characteristics, such as age and gender, educational attainment, personal feeding habits/dietary preference, feeding habits/dietary preference adopted for the pets and child/children, and choice of diet fed to the pets and child/children (Table 1). Although participant selection sought diversity across these characteristics, the pool of questionnaire respondents was skewed toward individuals with relatively high educational attainment. Consequently, these characteristics were reflected in the final interview sample and should be considered when interpreting the transferability of the findings.

**Table 1:** Characteristics of interview participants.

Variable	*n* (%)
1. Please identify your age in years	
18–24	1 (6)
25–35	8 (47)
36–45	7 (41)
46–55	1 (6)
2. Which of the following best describes the area you live in?	
Urban (densely populated inner town or city)	6 (35)
Suburban (less densely populated outskirts of a town or city)	6 (35)
Rural (based in the countryside)	5 (30)
3. What is your highest level of education?	
Up to completion of secondary education	2 (12)
Post secondary education	1 (6)
Undergraduate or equivalent level (e.g., Bachelor’s degree)	8 (47)
Postgraduate or equivalent level (e.g., Master’s / Doctoral degree)	6 (35)
4. Are you employed or involved in the veterinary healthcare field or pet animal industry?	
No	10 (59)
Yes, veterinarian or veterinary nurse	3 (17)
Yes, student of veterinary medicine, veterinary science or veterinary nursing	1 (6)
Yes, breeder	1 (6)
Yes, within an animal service (behaviorist/trainer, groomer, farrier, animal acupuncturist, kennel staff, veterinary receptionist, etc.)	2 (12)
Yes, within an animal industry (pet food, pet toys, pet products)	0
Yes, other	0
5. Are you employed or involved in the human healthcare or diet and fitness industry?	
No	13 (76)
Yes, healthcare professional – Doctor (generalist or specialist practitioner), Nurse, Midwife, Dentist, Pharmacist	1 (6)
Yes, student healthcare professional (as listed above)	0
Yes, within an allied healthcare service (e.g., physiotherapist, psychologist, paramedic, radiographer, dental hygienist/assistant)	1 (6)
Yes, within the diet and fitness industry (e.g., dietician/nutritionist, personal trainer/fitness instructor, health coach, health/diet-related sales representative).	2 (12)
Yes, other	0
6. What is your personal dietary preference?	
Omnivore (meat and plant-based diet)	9 (53)
Pescatarian (containing fish, but no other meats)	0
Vegetarian (avoiding all meat but including eggs, dairy and/or honey)	6 (35)
Vegan (free from all animal products)	2 (12)
Other	0
7. Do you follow an alternative diet? (Tick all that apply)	
Gluten-free or another specific food allergy or intolerance (e.g., lactose-free)	4
Intermittent fasting	5
Ketogenic diet (low carbohydrate, high fat)	1
Low carbohydrate diet	3
Low fat diet	1
Mediterranean diet (rich in vegetables, fish, fruit, grains, olive oil, and nuts, moderate dairy and minimal red meat)	3
Paleolithic (paleo) diet (avoiding all processed foods)	2
Other	0
N/A - I do not follow any alternative diet	8
8. As a parent or guardian, how many children aged 0–10 years do you have responsibility for feeding?	
1	7 (41)
2	5 (29)
3	2 (12)
4	1 (6)
5	1 (6)
6 or more	1 (6)
9. What dietary preference do you adopt for the child/children?	
Omnivore (meat and plant-based diet)	9 (53)
Meat based	3 (17.5)
Vegetarian (avoiding all meat but including eggs, dairy and/or honey)	3 (17.5)
Vegan (free from all animal products)	2 (12)
Other	0
10. Do you feed the child/children any of the following alternative diets? (Tick all that apply)	
Gluten-free or another specific food allergy or intolerance (e.g., lactose-free)	4
Intermittent fasting	2
Ketogenic diet (low carbohydrate, high fat)	2
Low carbohydrate diet	0
Low fat diet	1
Mediterranean diet (rich in vegetables, fish, fruit, grains, olive oil, and nuts, moderate dairy and minimal red meat)	4
Paleolithic (paleo) diet (avoiding all processed foods)	1
Other	0
N/A - I do not feed the child/children any alternative diet	8
11. How many dogs and/or cats do you have responsibility for feeding?	
Dogs – none	6
Dogs x1	8
Dogs x2	2
Dogs x3	1
Cats – none	4
Cats x1	8
Cats x2	4
Cats x3	1
Cats x4	0
12. What dietary preference do you adopt for each of your pet(s)?	
Omnivore (meat and plant-based diet)	5 (29)
Meat-based	10 (59)
Vegetarian (avoiding all meat but including eggs, dairy and/or honey)	2 (12)
Vegan (free from all animal products)	0
Other	0
13. What type of diet do you feed to your pet(s)? (Tick all that apply)	
Commercial cooked diet (dry/kibble or wet/canned/tinned/sachet)	11
Home-prepared cooked diet	3
Commercial raw meat diet	3
Home-prepared raw meat diet	2
Combination of commercial and homemade diet (cooked)	4
Combination of commercial and homemade diet (raw meat)	1
Freeze-dried or rehydrated commercial pet foods	1
Grain-free diet	4
Human-grade fresh or frozen seafood	1
Novel protein diet (e.g., insect based)	1
Prescription/therapeutic diet (e.g., food allergy, liver-care, kidney-care etc.)	1
Raw bones	0
Tailor made diet (individualised to your pet)	1
Weight loss diet	0
Other (please specify) __________	0

Consistent with an interpretive thematic analysis approach, sample adequacy was judged by the depth and diversity of the dataset and its ability to support meaningful interpretation, rather than by the achievement of thematic saturation (105, 107, 108).

### 2.5 Interviews

Interviews were held between 4 September and 14 November 2024. A £15 Amazon voucher was offered as remuneration for participation. A semi-structured interview script (Supplementary File 2) was developed by the primary researcher (R. L.) and underwent expert review by the co-authors (S. F., P. Y. and J. R.) and an experienced qualitative researcher from the University of Glasgow. Feedback focused on alignment with the research objectives, neutrality, question order and flow, and coverage of key concepts. Following revision, the interview guide was reviewed by five purposively recruited non-participant caregivers who met the study inclusion criteria and were known to R. L. to assess the clarity, comprehensibility, and acceptability of the questions. Based on this feedback, minor amendments were made to improve the readability and wording of the interview guide, while preserving the content and intent of the questions. Key areas included factors informing caregivers’ diet decision making and feeding practice, perceptions of the importance of nutrition, attachment to dependents, and beliefs pertaining to minimally processed and ultra-processed foods. Questions provided structure across interviews but were open-ended to allow flexibility for caregivers to elaborate freely without specific prompting.

Interviews were conducted online via video-conferencing software (Zoom.com), with only the participant and the primary researcher present. Audio-recorded informed consent for participation and recording was obtained from each participant prior to the interview. Interviews typically began with a question about feeding management of dependents across the course of an average week. In accordance with Vidgen and Gallegos (109) and Wijayaratne et al. (51), this provided a foundation on which to obtain information. Interviews ranged from 27 to 66 min in duration, averaging 47 min, and were transcribed by R. L. Any identifiable data, such as the names of participants, family members and pets, were anonymized before uploading transcripts to NVivo 14.24.3 (7) for analysis. At the time of interview, participants were offered the opportunity to review their interview transcripts as a form of respondent validation (110) to verify the accuracy of the interview record and clarify any factual inaccuracies or ambiguities. This process was intended as transcript verification rather than validation of the developing analysis (111), however this opportunity was declined by all.

### 2.6 Data analysis

Interview data was interpreted by R. L. using interpretive thematic analysis (112) (Table 2). This method acknowledges the phenomenological approach to understanding the experiences of individuals and encourages deeper analysis and sense making. The semi-structured interview script served as an initial organizing framework for data analysis, with data segmented according to the responses to each question (113). Inductive immersion enabled familiarization with the data and was achieved by the primary researcher (R. L.) who listened to the recordings while reading the transcripts. This process enabled familiarization with the data and was one of nine heuristics: learning as you go; inductive immersion; annotating; coding; sensitizing; seeking coherence; memoing; iterating; and being reflexive. This non-linear process enabled interpretive analysis of the data and facilitated an open-minded engagement with the material (112).

**Table 2:** Summary of the analytical stages of the study.

The nine heuristics of interpretive analysis (112)	Application to the current study
1. Learning as you go	Notes and memos were made during each interview.
2. Inductive immersion	After each interview, the transcript was read alongside listening to each recording. This facilitated self-familiarization with the data.
3. Annotating	NVivo (version 14) was used, and this enabled notes to be made.
4. Coding	Open coding was conducted using NVivo. Text passages were highlighted and annotated with simple and precise codes to enable easy discovery and the establishment of connections.
5. Sensitizing	External ideas and literature were considered to facilitate sense-making.
6. Seeking coherence	Codes were examined and initial connections and themes were identified.
7. Memoing	Connections were drawn between elements and longer notes were produced to help make sense of codes and the generation of themes, subthemes and overarching themes.
8. Iterating	The steps above were repeated to prevent hasty assumptions from being made.
9. Being Reflexive	Presuppositions (personal motivations, experiences and emotions) were acknowledged and the potential impact of these on data analysis were considered.

An inductive, ‘data-driven’ approach to coding was performed, with codes developed from the data (114). Although interpretation was inevitably shaped by the researchers’ positionality, reflexive discussions throughout the analysis helped critically examine assumptions and ensure that themes remained grounded in participants’ accounts (115). R. L. conducted the primary analysis, which was an iterative and recursive process, moving between semantic coding of participants’ explicit accounts and latent coding to interpret the underlying assumptions, ideas and meanings that informed those accounts. Initial inductive coding with the identification of semantic and latent codes was performed, followed by the iterative development of themes and subthemes, and the identification of exemplar quotes (112). S. F. reviewed the developing themes, contributed to discussions regarding their interpretation, and collaboratively refined and co-developed the final thematic framework. Rigor and validity of the findings was achieved through ongoing reflexive practice, data immersion and interpretive analysis, with sense-making beginning soon after data collection (112). An audit trail of all stages of analysis was kept to further maximize the trustworthiness of findings (116–118).

The reported findings relate specifically to the factors that influence caregivers’ diet choice and feeding practice, perceptions of the importance of nutrition and level of attachment to dependents. Findings related to caregivers’ claims and motivations for the feeding and avoidance of minimally processed and ultra-processed food will be reported in a subsequent publication. Illustrative quotes were labeled according to the unique identification number of each caregiver (C).

Themes illustrate the range of psychosocial and contextual influences on the four key research areas: caregivers’ diet choice, feeding management, perceptions of the importance of nutrition, and level of attachment to dependents. A conceptual overview of each theme and its associated subthemes is included below. Exemplar participant quotes have been provided verbatim, with square brackets enclosing words added or modified for context and to de-identify data and remove reference to names of brands and food manufacturers. Ellipses enclosed within parentheses represent the omission of irrelevant words and have been used to maintain integrity and focus.

## 3 Results

Four themes, with associated subthemes (Table 3; Figure 1), were identified in relation to the study objectives. Although the themes were analytically distinct, they were interconnected, with each capturing a different dimension of caregivers’ feeding practices. Theme one established feeding as a caregiving responsibility, with nutrition positioned as fundamental to health and wellbeing. Theme two explores the psychosocial influences underpinning dietary decision-making, including knowledge, beliefs, habits and social norms. Theme three examines how caregivers’ nutritional ideals are negotiated in practice in response to the realities, preferences and needs of those being fed. Finally, theme four considers how feeding is situated within caregivers’ relationship with children and companion animals, highlighting the role of attachment, family membership and shifting priorities across species. The findings move from caregivers’ conceptualization of feeding to the psychosocial influences shaping feeding practices, the negotiation of those practices, and finally their relational and interspecies context. Together, these themes illustrate how feeding decisions were shaped by the interplay between moral values, practical negotiation, relational bonds, and psychosocial influences.

**Table 3:** Theme summary table.

Themes		Subthemes		Main take home messages*
1	The Responsibility of Feeding: Health, Wellbeing and Duty	1.1	Nutrition as fundamental to health	Nutrition is important Caregivers prioritize diets that support health and wellbeing
		1.2	Feeding as a core caregiving responsibility within everyday constraints.	Provision of balanced nutrition is part of responsible caregiving. Pet feeding is characterized by greater uncertainty.
2	Nurturing Across Species: Psychosocial Determinants of Feeding	2.1	Norms, habit and tradition	Habit and tradition shapes feeding routines and dietary choices for children and pets.
		2.2	Caregivers’ knowledge and personal beliefs	Caregivers’ feeding decisions are shaped by trust, informal advice, and knowledge gaps, often constrained by practical realities and personal values.
		2.3	Food, family and affection: The social dimensions of feeding	Mealtimes with children are socially structured and value-driven. Pet feeding is more informal and emotionally motivated.
3	Negotiating feeding: balancing nutritional ideals with responsible and responsive feeding	3.1	Translating nutritional ideals into feeding decisions	Balanced nutrition is valued across species. Feeding decisions are shaped by practical and emotional considerations.
		3.2	The bidirectional dynamic of the feeding relationship	Feeding children and pets is a relational and emotionally complex process in which dietary goals are continually adapted.
4	Redefining Kinship: Caregivers Attachment to Pets and Children	4.1	Pets as Family Members and Sources of Companionship	Emotional attachment to pets is strong but dynamic, and shaped by daily caregiving routines and responsibilities.
		4.2	Shifting priorities and a reordering of the ‘pecking order’	Children restructure caregivers’ attachment, resulting in deprioritization of pets within the household hierarchy.

* The main take home messages provide concise summary statements of the key insights represented by each theme and subtheme. They are intended to aid interpretation and do not represent an additional stage of analysis beyond the thematic framework.

**Figure 1:**
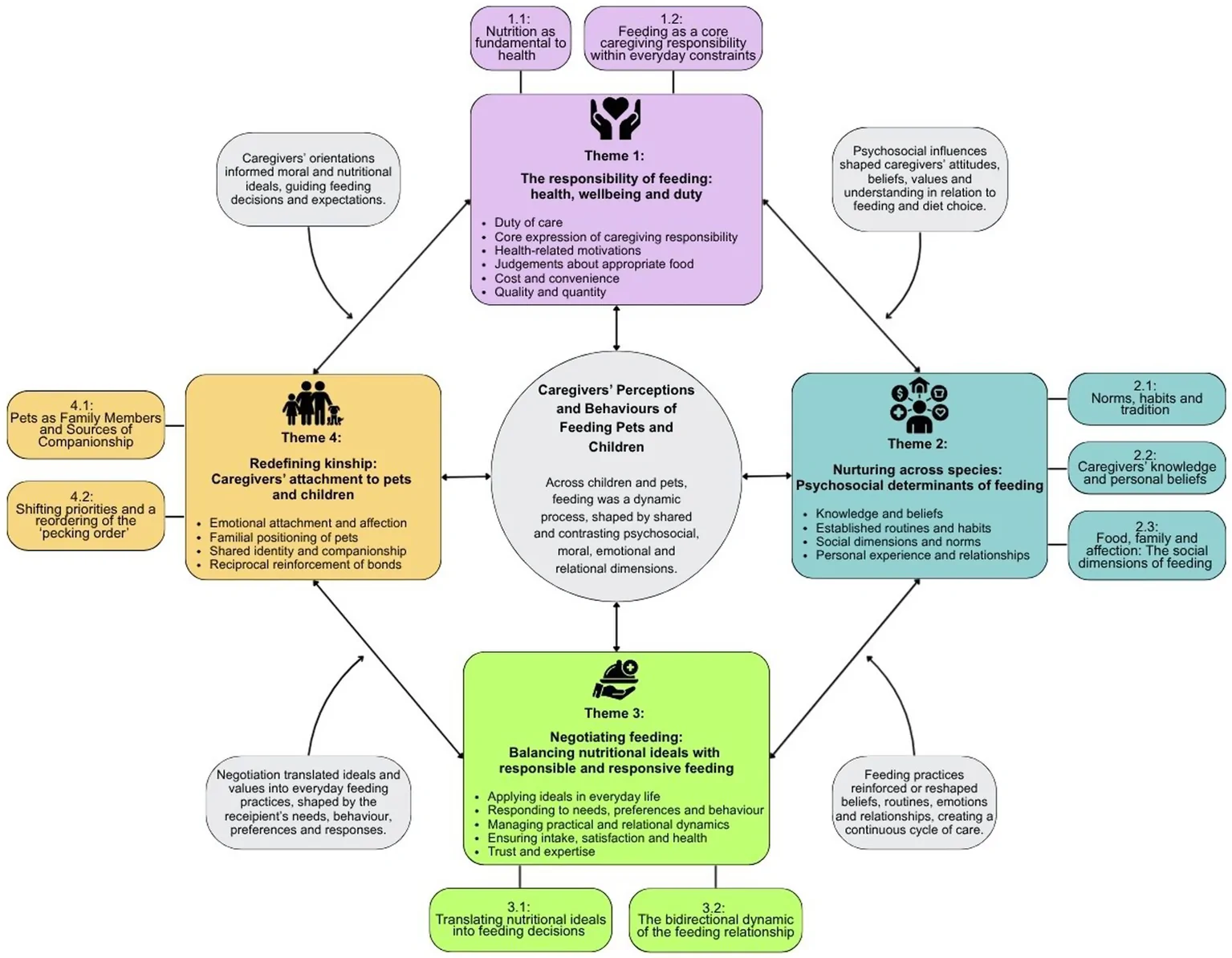
Thematic framework of caregivers’ perceptions and behaviors in feeding children and pets. Image: Canva.com and Flaticon.com. This figure has been designed using Canva and resources from Flaticon.com.

### 3.1 Characteristics of study participants

Of the 17 participants, 53% (*n* = 9) had children aged 0–5 years and 88% (*n* = 15) had children aged 6–10 years, with some having children in both age groups. A greater proportion of females (59%, *n* = 10) than males (41%, *n* = 7) were interviewed. There was near even divide between the <35 (*n* = 9) and >36 (*n* = 8) age groups and in the proportions of individuals residing in urban (*n* = 6), suburban (*n* = 6), and rural (*n* = 5) areas. Most participants (82%, *n* = 14) were graduates with seven and four participants employed or involved in, respectively, the veterinary or pet industry, and the human healthcare or diet/fitness industry.

### 3.2 Theme 1: the responsibility of feeding: health, wellbeing and duty

Diet choice and feeding practices were framed as an important duty of care negotiated within the constraints of everyday life, with nutrition viewed as fundamental to health and wellbeing, as well as a core expression of caregiving responsibility.

#### 3.2.1 Subtheme 1.1: nutrition as fundamental to health

Findings converged with participants unanimously viewing nutrition as extremely important for humans and animals and vital to maintaining health. An optimal diet was perceived as being directly linked to wellbeing, growth, development, happiness, longevity and performance, while poor diet was associated with illness or poor condition/outcomes.

*I think it’s important for both animals and people to grow healthily, you have to have the right nutrition*. (C57, female caregiver to two children and one dog)

Caregivers’ choice of pet food was influenced by factors arising from their pet’s biology, food response, and the outcomes they wanted to achieve, for example, health and enjoyment. A desire to feed a balanced and nutritious diet was a key consideration for many caregivers, with one quick to highlight the cross-species importance.

*In the same way humans need nutrition and a balanced diet, pets also need a balance[d] diet* (C42, male caregiver to three children, two dogs and two cats).

The presence of underlying medical conditions was another key dictator of diet choice, particularly for pets.

*[Cat] had digestive problems as a young cat so the vet put us on, [current brand], and I’ve never changed it, only because I do not know if his digestion could take it.* (C7, female caregiver to one child, one dog and one cat)

*We went through hundreds of brands because we want him to have wet food, but he’s got a very sensitive stomach with wet food, so he’s vomited on so many brands that, actually, we are now completely limited to [current brand] and there’s a very specific box that we have to buy.* (C9, female caregiver to one child and one cat)

#### 3.2.2 Subtheme 1.2: feeding as a core caregiving responsibility within everyday constraints

Caregivers described feeding as an important personal and moral responsibility that extended beyond simply providing food to actively managing what, when and how much their children and pets consumed. However, the enactment of this responsibility was shaped by everyday practical and financial constraints. Many felt it important to make conscious efforts to ensure quality and balance in the food they chose to feed. Caregiving duties included monitoring food intake, selecting higher-quality or fresh foods, restricting certain items (e.g., fizzy drinks), and choosing diets that aligned with personal or family values.

*I’m always on about, “Has she had fruit? (…) Has she had vegetables?” (…) and with the dogs as well, and the cats, like a good quality mid-range, or if you can afford better, but I think a balanced diet is super important.* (C13, female caregiver to one child, one dog and three cats)

*I think it’s more just from a parental side of it, wanting to make sure I’m giving them the best-rounded kind of diet*. (C81, female caregiver to two children and two cats)

Time and convenience shaped how caregivers enacted their feeding responsibilities. Although many aimed to provide home-cooked, nutritionally balanced meals for children, work and household demands sometimes led them to prioritize quick foods requiring minimal preparation, particularly during the week when there were more time constraints.

*Sometimes it’s quite quick things, because I work quite late in the evenings [whereas] at weekends, I can be a bit more adventurous because we have more time.* (C82, female caregiver to one child and one cat)

*When we get home. It’s a bit of a clamor of what’s for tea? And everyone wants everything as quickly as possible.* (C70, female caregiver to one child and one dog)

*I am very, very busy, and I do not have time to cook more than one meal so (…) this way I get to make one dinner, so they just eat what I eat.* (C66, female caregiver to three children and two cats)

Economic constraints were further identified as a key determinant for the majority of caregivers.

*Obviously the big one in the current climate is finance, very much.* (C13, female caregiver to one child, one dog and three cats)

*I do go for cheap things, you know, since I’m not really that financially stable.* (C45, male caregiver to one child, three dogs and one cat)

Specifically for pet caregivers, cost was cited as a reason for avoiding dietary variety.

*It is expensive, you do not want to go and buy something that they are not going to eat.* (C69, female caregiver to two children and two cats)

*I stick to what I know he likes so that it’s not wasted, and so that he eats it.* (C82, female caregiver to one child and one cat)

Cost was also considered an indicator of quality.

*We do not end up buying the cheapest stuff. We’re very much, it’s mid-range, really and it’s rarely on offer these days (…) I would not buy the cheap tins that you can get, because they are a bit gross (…) it just looks lower grade to me*. (C69, female caregiver to two children and two cats)

*We’re not feeding them premium ones [diets], and we have gone through phases where we have tried, but we genuinely just cannot keep up with the cost of them*. (C81, female caregiver to two children and two cats)

Caregiving responsibility also extended to regulating the amount of food consumed. Limited diversity was reported in pet feeding methods, with most caregivers using a standard bowl. One caregiver utilized an automatic feeder system for her cat to help avoid overfeeding and “*unwanted weight gain*” as well as for the lifestyle gain, “*it’s easier for my partner and I to manage part of his feeding if we’re not really having to manage it at all*” (C9). Portion management for children was often based on subjective estimation, with a level of input by children’s cues.

*Normally, I just sort of put the portion on a plate that looks about right and I mean, I guess normally I do about a 3rd protein, a 3rd vegetable, a 3rd rice, or whatever.* (C66, female caregiver to three children and two cats)

*We generally have quite large portions, so I do try and manage it that he eats at least half of it [but] it’s really difficult with children to manage anything (…) so it’s probably more child-led*. (C7, female caregiver to one child, one dog and one cat)

Comparable reliance on subjective estimation of pet feeding amount rather than precise measurement of food portions was reported, underscoring the ambiguity caregivers experience when attempting to interpret feeding guidelines.

*It does say on the back of the packet of the pouches how much you should give them, based on like how big they are. I think he is quite a big boy. He′s quite chubby (…) I kind of just guess if I’m honest* (C82, female caregiver to one child and one cat).

Caregivers perceived their misjudgments in appropriate portion sizes and in recognizing optimal bodyweight as contributing to undesirable pet weight gain. Accounts were frequently associated with feelings of guilt and humiliation, and this was a driver for the modification of feeding practice.

*He just got fatter and fatter and fatter like it was, it got to be a bit embarrassing because I used to take it to the vets, and I think he’d lost weight and I was cutting it down to half as well, and he would have put on like 4 kilos (…) [now] I like the fact that I can see he’s losing weight. And I’m going to the vets tomorrow and I know I’m not gonna have ‘the talk’ is really good.* (C7, female caregiver to one child, one dog and one cat)

*The guilt of letting your dog get fat is terrible. It’s so easy, and it [the weight] goes on so gently that you do not really notice (…) that was a bad parent moment for me, a bad pet parent moment.* (C70, female caregiver to one child and one dog)

Notably, results contrasted with caregivers failing to describe the same degree of uncertainty, self-blame, or subsequent adjustment of feeding behaviors in children.

### 3.3 Theme 2: nurturing across species: psychosocial determinants of feeding

Across accounts of feeding children and companion animals, caregivers’ feeding practices were shaped by a range of psychosocial influences extending beyond nutritional considerations. These influences were often embedded within everyday family life, reflecting the ways in which feeding practices are learned, negotiated and adapted through personal experience, relationships, and practical circumstances.

#### 3.3.1 Subtheme 2.1: norms, habits and tradition

Many of the caregivers interviewed spoke of habit and tradition, with some reflecting on their own childhood experiences and the impact this had on their own parenting.

*What my parents did for me, that brought the best out of me [and] could actually yield a very great result in future in them, as well*. (C67, male caregiver to five children, one dog and one cat)

*I always valued mealtimes growing up (…) that’s a nice family time, you can sit and talk.* (C81, female caregiver to two children and two cats)

One first time mother spoke of the influence that dietary norms has had on her feeding behavior and her decision to feed her toddler three meals per day, despite her and her partner choosing not to eat breakfast.

“*It’s just the way that my friends have done it. And yeah, just fallen into that trap of breakfast, lunch and dinner*” (C9, female caregiver to one child and one cat).

In general, however, caregivers’ routine and eating schedules remained largely the same as before having children, with accommodations made where necessary, particularly in relation to timing.

*We eat, particularly dinner, considerably earlier, or quite a bit earlier than we’d be used to. But other than that, (…) this is what we did before, so we’ll continue doing it now*. (C6, male caregiver to two children and one cat)

*You do what you have always done, I suppose, and you do adapt because they are children and they are fussy*. (C69, female caregiver to two children and two cats)

Comparably, habit and tradition were both influential factors in the food choice and feeding management of pets.

*I chose it [the diet] because it worked for [previous dog], but it definitely did not work for [current dog].* (C7, female caregiver to one child, one dog and one cat)

*It’s what I’ve always done, and whenever we have had cats when I was little, that’s what my mum did. So I just kind of did the same thing*. (C82, female caregiver to one child and one cat)

#### 3.3.2 Subtheme 2.2: caregivers’ knowledge and personal beliefs

Pet caregivers’ nutritional knowledge was shaped by the information sources available to them and interacted with their personal beliefs and values to influence feeding decisions. While some caregivers sought advice from friends, family, or online resources, others highlighted the limited availability of educational guidance for companion animal nutrition compared with child feeding. One pronounced divergence was the contrast in structured guidance available for infant feeding with the absence of comparable educational resources for companion animals. For one caregiver, this contributed to reliance on retail availability and assumed adequacy of commercial pet foods, rather than deliberate or informed decision-making.

*When you have children, (…) a lot of the books on bringing up babies and weaning have got information about food and where you should start and how you should do it. I never got a book with the cats. I never stopped to think, how am I going to go about this? You just know that shops sell cat food, and you go out and buy it*. (C69, female caregiver to two children and two cats).

Caregivers typically selected a diet for their children that mirrored their own, largely for convenience and personal beliefs. For plant-based diet consumers, food choices were framed as an extension of ethical values, which were routinely embedded within family feeding practices. However, caregivers remained accepting and supportive of potential future dietary changes. This positioning highlights the interplay between value-driven feeding practices and a desire to foster independent dietary decision-making.

*I wasn’t about to change my cooking habits to, you know, introduce something that I was not going to eat either*. *But I think they totally accept that they are happy not eating meat*. (C69, female caregiver to two children and two cats)

*Obviously, the vegan aspect is just morally we, that’s our kind of views and we have brought the kids up, and they, I mean, if they get to a point where they do not want to be vegan, we are more than happy for them to make that choice, and they can, we can support that, and that’s absolutely fine*. (C81, female caregiver to two children and two cats)

Caregivers’ accounts illustrated diverse approaches to pet feeding, with many drawing on their understanding of companion animal nutritional needs alongside personal beliefs, values, and available sources of information. After observing his cat’s weight loss, and at the recommendation of friends, one caregiver “*chose to feed milk and fish rather than vegetables*” (C39), demonstrating knowledge deficits in relation to cats’ nutritional requirements. Caregivers who favored a plant-based diet actively negotiated tensions between their personal dietary identities and their understanding of species-specific nutritional requirements.

*We had the cats before we went vegan, so they were on [commercial meat-based diet], and that’s what they were used to and so (…) we just did not think it was fair to suddenly, like cats, notoriously, they are carnivores, they are meat eaters. You can get vegan cat food. We looked into it, we researched it, and you can get it, but I did not feel as comfortable about doing that with them than I did [the children].* (C81, female caregiver to two children and two cats).

One homemade diet feeder felt variety improved his dog’s appetite and helped reduce over-reliance on one brand or flavor (C35) but, in general, variety wasn’t regarded as important for pets as it was for children. Another caregiver commented that his dog needed a *“more limited and specific diet than humans”* and prioritized the feeding of a diet that is safe to consume and which meets the pet’s nutritional needs (C42). Another expressed reservation that “*by mixing it up, you might end up doing the wrong thing*” but voiced concerns about feeding the same diet long-term (C6).

*I worry a little bit about, because we have had him on one consistent diet for a long time, you know, every so often, as much as I do have a degree of trust in the nutritionists and feed companies that they do search for a balanced diet*. (C6, male caregiver to two children and one cat)

Awareness of life stage feeding was apparent, but not all caregivers were knowledgeable of its importance or the varying nutritional needs at each phase of life. Provision of life stage-specific nutrition posed challenges within multi-pet household settings.

*It’s too difficult [feeding different diets], because [youngest cat] will always eat [eldest cat]'s food (…) I do not know what the difference is [between kitten and senior food]. I wonder if the senior stuff is actually just easier for them to chew, because I do not think the nutrients are any different.* (C69, female caregiver to two children and two cats)

*We put kitten food down and adult food down, and they graze (…) there’s possibly some cross feeding across the way, because we just would not be able to separate them.* (C13, female caregiver to one child, one dog and three cats)

Other considerations included diet availability and the ability to “*secure a consistent diet*” (C6), as well as delivery and storage (C70) and environmental impact and sustainability (C13).

#### 3.3.3 Subtheme 2.3: food, family and affection: the social dimensions of feeding

The ideal of family meals is reinforced by social norms and is an opportunity for caregivers to pass on family values and traditions, strengthen family relationships and promote healthy eating habits. Meals were commonly considered an opportunity for social family time and eaten at a table to facilitate social eating, reduce the chance of choking and limit mess. Yet, as one caregiver commented, “*there is a lifestyle factor (…) does the child like eating where there is a social interaction or does it like eating alone?*” (C42).

On weekdays, eating schedules were, largely, dictated by the timing of children’s school and extracurricular activities. Whilst there was a general desire for shared mealtimes, this was often dictated by caregivers’ work commitments and dinner was the most consistently prioritized family meal.

*We try and encourage that we all sit down together, and we all stay at the table till everybody’s finished, or they have had enough.* (C57, female caregiver to two children and one dog)

*Well, if [Partner]'s working away then obviously, he’ll have his [dinner] later. But we always eat dinner together at weekends. (…) we do not necessarily have breakfast or lunch together, but we always eat dinner together.* (C69, female caregiver to two children and two cats)

By contrast, shared mealtimes with pets were uncommon, representing one of the clearest divergences from child feeding practices, with only one caregiver reported a preference for social eating with her pet.

*When I dish up for the family, I also do the dog food (…) I like to feed us at the same time. Yeah, it’s just my, my preference. She should not wait till after we [eat]. I think that’s an outdated idea now of the pecking order of food and stuff, you know.* (C70, female caregiver to one child and one dog)

Pet feeding schedules were primarily shaped by convenience and caregivers’ lifestyle and working schedules.

*I think if I did not work, she’d probably get 3 meals a day like we do, but it’s just not convenient, because I’m at work so (…) twice a day has just worked for us, and that’s the only reason.* (C57, female caregiver to two children and one dog)

*At least one of us is in all day. They’re getting fed more frequently, but smaller amounts than if we were both out working.* (C69, female caregiver to two children and two cats).

*It’s always just been a thing that he kind of gets fed little and often rather than just in the morning, and then at night, because yeah, I’m in and out*. (C82, female caregiver to one child and one cat).

Beyond the practical organization of mealtimes, caregivers also described the social and emotional functions of food. Treats and snacks were frequently used to express affection, reward behavior, and reinforce relationships with both children and companion animals. Treat provision in pets emerged as a psychosocially driven feeding behavior, often motivated by emotional bonding, anthropomorphism or in response to specific pet behaviors, rather than nutritional considerations.

*I would not say every day [he gets treats]. He′s a little bit older now, I guess if he was making more of a racket, we would give it to him just to shush him.* (C9, female caregiver to one child and one cat)

*She also does get some treats with us and from us as well, because she’s a pet, you know, and it’s nice (…) to be able to give her those little bits off the plate because it involves her in the family.* (C70, female caregiver to one child and one dog)

In contrast, treat and snack provision for children was typically structured, moderated and context dependent. Caregivers commonly framed treats as occasional and earned, often linked to specific times (e.g., weekends), events (e.g., meals out, holidays) or behaviors (e.g., eating main meals or maintaining a balanced diet). Snacks, more broadly, were managed with varying degrees of control and were influenced by the restrictions imposed by schools and nurseries. Caregivers attempted to limit snack frequency or type to protect health and appetite for main meals, though this was sometimes challenged by children’s requests, preferences, or “*grazing*” tendencies. In response, some caregivers emphasized healthier snack alternatives (e.g., fruit or homemade options) or portion control strategies. Others described the use of snacks as tools for behavior management, distraction, or social participation.

*I’ve stocked up my freezer with [homemade] snacks (…) I’ve made them, I know what goes into them, I feel quite comfortable giving them to him and then other snacks potentially would be when we are out and about, and he’s getting cranky, or he needs distracting.* (C9, female caregiver to one child and one cat)

*They get those little mini snacks in a box that they can share with the other kids as like a social thing.* (C66, female caregiver to three children and two cats)

### 3.4 Theme 3: negotiating feeding: balancing nutritional ideals with responsible and responsive feeding

Convergence between caregivers was noted in relation to feeding, which emerged as a form of care in which health goals were continually balanced with responsiveness to children’s and pets’ needs and behaviors. Caregivers strived for nutritional ideals, whilst managing the practical and relational dynamics of feeding in ways that ensured intake, satisfaction, and ongoing health. In contrast to theme one which recognizes feeding as an important caregiving responsibility, this theme highlights the challenge of reconciling caregivers’ ideals with the preferences, behaviors and needs of those being fed.

#### 3.4.1 Subtheme 3.1: translating nutritional ideals into feeding decisions

Caregivers operationalized health goals and made dietary judgements for their dependents according to nutritional knowledge and observable cues, including assessment of body condition, skin condition (children) or coat quality (pets), digestion, activity levels, and general demeanor. For pets in particular, caregivers’ trust in commercial diets, brand reputation, effective marketing and veterinarian endorsement often substituted for a detailed understanding of ingredients.

*When you look at your animal that is healthy, you actually know, without being told.* (C32, male caregiver to six children, one dog and one cat).

*For my children I would always make sure that they have got plenty of fruit and vegetables, plenty of water, like I do the dog’s always got fresh water, that sort of thing. But yeah, maybe I should be treating my dog exactly as I treat my children, because I just assume the commercial diet will have all the nutrients and things that she needs to keep her healthy.* (C57, female caregiver to two children and one dog).

Whilst some caregivers voiced skepticism toward nutritional adequacy, most expressed trust and convenience in the feeding of commercial pet foods and their ability to provide a balanced diet.

*Nutrition for me has never come into it with the cats, you know, this is cat food, so this is what I buy, and I’ve never thought beyond that (…) It’s meat and cats eat meat (…) I’m sure the percentage of meat in some of this stuff is fairly minimal, but they like it*. (C69, female caregiver to two children and two cats).

*If you go for a commercial cat food, you know they are getting the right nutrients (…) so it’s a nice, easy option, I suppose*. (C81, female caregiver to two children and two cats).

Despite a desire for their children to be “*as healthy as they possibly can*” (C57), some caregivers described the challenge of catering for “*picky*” eaters (C7, C81) and those with selective appetites (C69). Perceptions of a healthy, balanced diet often included fruit and/or vegetables as an essential component. Yet caregivers reflected on the challenges associated with provision of these foods, with many describing the need to “*hide*” (C7, C57) and “*disguise*” (C69) nutritious ingredients within meals.

*I have to think about his meals as in what can I hide that is really good for him amongst the wholesome meal that he enjoys.* (C7, female caregiver to one child, one dog and one cat)

*I always try and hide vegetables in it like grating up carrots and chopping courgettes and onions, and everything really small.* (C57, female caregiver to two children and one dog)

The emotional impact of diet selection was also apparent with one caregiver sharing her concerns about being “*so strict that they sort of rebel against us when they’re older*” (C57). Whilst another was eager to ensure his children’s happiness, commenting that they should “*go for what they actually want (…) and what will make them happy*” (C32).

Caregivers appeared to judge their pet’s food consumption and behavioral response as an indicator of health, happiness and contentment.

*He eats it. He′s happy, and he wants it*. (C6, male caregiver to two children and one cat).

*I just love giving fish and meat because I noticed that’s what made the cat happy*. (C39, male caregiver to two children and one dog)

*When she’s not well, she does not eat. She just refuses.* (C57, female caregiver to two children and one dog)

#### 3.4.2 Subtheme 3.2: the bidirectional dynamic of the feeding relationship

The complex interplay between caregivers’ feeding practices and dependents’ cues, preferences and eating behaviors were key influences in food choice. Yet one of the clearest divergences between child and companion animal feeding concerned the capacity of dependents to shape caregivers’ feeding behaviors. While caregivers responded to both children’s and pets’ behavioral cues, children’s preferences and food refusal were typically interpreted as expressions of developing autonomy requiring ongoing negotiation.

*If she really does not like something, and if it’s something for the first time, we’ll try and encourage her to eat it (…) the more you try and force them, the more they are going to hate it.* (C13, female caregiver to one child, one dog and three cats)

*There are times they may not want to eat, and I try my best to convince them. You know, they are kids, at times you have got to plead with them to eat the food.* (C32, male caregiver to six children, one dog and one cat)

*We do not force them to eat the food to finish their plate, but we want them to eat enough that they are not going to say within an hour, “I’m hungry” because we know that’s what happens if they do not.* (C57, female caregiver to two children and one dog)

In contrast, pets’ behavioral cues influenced feeding practices to a lesser extent through emotional and anthropomorphic responses, and were more commonly interpreted in relation to appetite, routine, or health than as behaviors requiring ongoing negotiation.

*The meowing can be tricky, especially during nap times (…) I wonder if he is aware that there are certain parts of the house that I would respond to him quicker.* (C9, female caregiver to one child and one cat)

*I’m pretty strict with the dog mainly because I let her get overweight and we had to go on a weight loss journey with her and have some very expensive weight loss, diet foods, which taught me my lesson*. *Do not let the dog get fat and it’s basically because she’s got the big brown eyes.* (C70, female caregiver to one child and one dog)

Notably, participants also described variation between caregivers, with pets appearing to modify their food-seeking behaviors according to who was feeding them. Caregivers perceived that pets learned which household members were more likely to respond to begging or provide larger portions, suggesting that feeding interactions were relational and shaped by reciprocal behavioral reinforcement.

*My husband’s worse than me (…) when I see how much he puts in the bowl, [it] is more. He does not measure out as accurately as I do, so he’ll just eyeball it, and I’m like,* ‘*that’s too much*’. (C57, female caregiver to two children and one dog).

*They pester [Partner] a lot more than they pester me, because they know he gives in and feeds them. So you know, day to day, I think if it was just me here, they would get a lot less than they actually do*. (C69, female caregiver to two children and two cats).

Despite caregivers’ desire to feed a balanced and healthy diet, children were often influential in shaping food selection. Accounts described the use of negotiation and compromise to ensure intake, sometimes at the expense of nutritional quality. This dynamic was accompanied by feelings of disappointment and dissatisfaction, reflecting the tension between caregivers’ dietary ideals and the practical realities of feeding.

*There’s probably a balance between feeding a balanced diet, but also trying to get something they will eat (…) There comes a point when you do probably start compromising on the content just to get something in.* (C6, male caregiver to two children and one cat)

*I’ll always start with the healthy option, and then we end up bartering*. (C69, female caregiver to two children and two cats)

Children’s influence on food choice was not always negative, with some caregivers reflecting on the positive impact on their own diets.

*We have within the last month really tried to change our habits, because we are becoming more aware that [Child] is becoming more aware about what we are eating. So we are making a lot of fresh food each night*. (C9, female caregiver to one child and one cat)

As with food choice, decisions regarding mealtimes were influenced by children’s eating habits.

*You cannot let your children dictate absolutely everything about your lifestyle and habits, you know, there’s a certain balance of neutral learning. The irony is, of course, they totally do, but we like to keep some level of control, I guess, somewhere, perhaps at some mealtimes.* (C6, male caregiver to two children and one cat)

When situations permitted, caregivers tried to provide children with the autonomy to make suitable choices. For example, one mother shared her desire to include her child in the decision making and give her choice because “*it’s her eating as well*” (C13) whilst another reflected on the positive impact this has on her child’s “*engagement of what she is eating*” and her likelihood to “*clean the plate*” (C70).

Caregivers reported their frustration when meals they had prepared were refused by children, with one reflecting on his feelings of anger, “*It’s one of the things that, it’s discouraged me, and it makes me angry at times*” (C32). Parental negotiation strategies were employed to circumvent child autonomy and promote food intake. These included distraction techniques such as the use of an audio story (C57), feeding homegrown produce (C13, C57) adding yogurt (C57) and the provision of snacks and gifts (C32). Whilst force-feeding was disfavored, opinion was sometimes divided on the appropriate course of action.

*I think it’s better when they are child led, so the child knows that, ‘Oh, I didn’t eat that dinner, so I’m now hungry’, that’s the lesson. Whereas sitting there for 3 h is not, you know, it’s not making anyone happy, is it?* (C7, female caregiver to one child, one dog and one cat)

*He′s kind of going on to a bit of a food aversion kick at the moment (…) we just kind of say, well, ‘this is what’s for dinner tonight and you can choose not to eat it, but this is what’s for dinner, and there won’t be any other options’*. (C9, female caregiver to one child and one cat)

The adequacy of food consumption of children was often judged over one or more days rather than per meal.

*If she’s hungry and she wants a snack, then I’ll give her the snack, and I’ll simply reduce her evening meal portion (…) so thinking about 24 hour food rather than each meal separately.* (C70, female caregiver to one child and one dog)

*What I’ve been reading is if he misses one meal every now and then. That’s okay. It’s the bigger scheme, so what he’s eating in a week.* (C9, female caregiver to one child and one cat).

### 3.5 Theme 4: redefining kinship: caregivers’ attachment to pets and children

Caregivers’ accounts pointed to a redefinition of kinship in which pets were often positioned as valued family members, embedded within everyday practices of care, affection, and companionship. Participants described strong emotional attachments to their animals, frequently framing them in familial terms and attributing to them needs, feelings, and relational significance. However, these attachments were not static.

#### 3.5.1 Subtheme 4.1: pets as family members and sources of companionship

This subtheme explores how caregivers described the emotional significance of pets, framing them as family members and valued companions. These narratives illuminate the meanings participants attached to their relationships with companion animals. Across participants, pets were consistently described as integral members of the family, offering companionship, emotional support, and affection.

*They are actually my best friends (…) and I think anything they want, I make provisions for them*. (C32, male caregiver to six children, one dog and one cat)

*She’s my furry baby (…) she’s a member of the family. I care about how she feels. I care about what she wants, what she needs.* (C70, female caregiver to one child and one dog)

Despite being considered deeply valued family members, the arrival of children typically reshaped caregivers’ attachment with their pets.

*Obviously, my child comes first, but in situations where I do not have to choose then I pick all of them.* (C35, female caregiver to one child and one dog)

*Because our days are so filled up, we probably do not sit down with him as much as we would do so, I guess I would say less attached to him [compared to our child] but I would not know how to scale that*. (C9, female caregiver to one child and one cat)

#### 3.5.2 Subtheme 4.2: shifting priorities and a reordering of the ‘pecking order’

Although participants frequently described pets as family members, these relationships did not necessarily translate into equal caregiving priorities. Attachment was dynamic and shaped by daily caregiving routines, household responsibilities, and the practical realities of caring for companion animals alongside children, with children’s needs typically taking precedence over those of pets. Whilst affection for pets remained strong, participants commonly described reduced time, attention and emotional capacity for them following the arrival of children, often accompanied by feelings of guilt.

*I am very fond of my cats. To be honest, they have been quite neglected since I had kids.* (C66, female caregiver to three children and two cats)

*They were our babies before we had our babies (…) we felt so guilty for such a long time, because we would not see them all day when the kids were around, and they’d only come out at night.* (C81, female caregiver to two children and two cats).

*He’s very cute but he’s probably the bottom of my pecking order (…) I do love him, but I find, sometimes I do feel like I have not got time for him, even though he’s only a cat, he does not take up a lot of time*. (C82, female caregiver to one child and one cat)

Caregivers’ sense of connection was further shaped by pets’ behavior, independence, and the child-pet relationship.

*They have fun together (…) He wants the pet to also go to school with him. He′s part of my family and they are also good friends*. (C42, male caregiver to three children, two dogs and two cats)

*I do not have the same level of care for them [cats] that I do for the children. But yeah, they are independent to a point, aren’t they? So, yeah, but they are very important.* (C69, female caregiver to two children and two cats)

## 4 Discussion

Nutrition plays a vital role in the development of humans and animals alike. Yet existing research has largely examined child feeding and companion animal feeding as separate fields of inquiry, with limited understanding of how caregivers navigate dietary decision-making across both contexts. The present study examined the factors influencing caregivers’ diet choices and feeding strategies for their child and dog or cat, their perceptions of nutritional importance, and level of attachment to their dependents. Although this study adopts a comparative One Health perspective, the intention is not to suggest direct equivalence between children and companion animals. Differences in developmental trajectories, nutritional requirements, physiological needs, decision-making capacity, and food environments necessarily shape feeding practices in each context. Accordingly, the comparison is conceptual, focusing on caregivers’ perceptions, experiences, and decision-making processes rather than implying direct comparability between species.

Findings help explain why people feed their pets and children the way they do, with caregivers’ strong commitment to nutrition as a marker of responsible and optimal care, coexisting alongside everyday constraints. The novel contribution of this study lies in its comparative examination of how caregivers’ beliefs, responsibilities, routines and perceptions of agency converge and diverge in shaping dietary decision-making for children and companion animals within the same household. The findings extend understanding of feeding as a relational and context-dependent caregiving practice. Taken together, the themes suggest that feeding was a dynamic process through which caregivers translated psychosocial beliefs and moral understandings into everyday practices, while simultaneously responding to the needs, behaviors and preferences of those being fed. These practices were further embedded within emotional and relational bonds, meaning that decisions about food were not simply nutritional decisions but also expressions of responsibility, care and attachment.

The findings should be interpreted within the cultural context in which the study was conducted. Participants were UK caregivers, and their accounts reflect Western conceptualizations of family, caregiving, and companion animal ownership, in which pets are increasingly viewed as family members and recipients of substantial emotional and financial investment. These norms may differ in cultural and geographic settings where companion animals occupy different social roles or where caregiving responsibilities and feeding practices are shaped by alternative cultural, religious, or socioeconomic influences. Consequently, the transferability of these findings should be considered in relation to similar contexts rather than assumed across diverse populations.

The Discussion is structured around the key themes and associated subthemes developed from the data. Each section presents the results within the context of existing literature, drawing on evidence from human and non-human species to provide a comparative perspective on feeding practices, caregiving dynamics and dietary decision-making.

### 4.1 Evaluating the importance of nutrition for good health and wellbeing

From a One Health perspective and, in accordance with other pet (119) and child-related (120–122) studies, caregivers in the current sample identified nutrition as critically important to the health of people and animals. The positive impact that a healthy diet has on mental and physical health and academic performance in children (123) was further recognized by some caregivers. Feeding and parenting are inextricably linked (47) and caregivers in the present study demonstrated a strong sense of responsibility for the health and wellbeing of the children and pets in their care, particularly through their role in shaping diet and feeding behaviors. Consistent with previous studies, (31, 122, 124, 125) some child caregivers also identified themselves as dietary role models for children. The parallels between feeding children and pets suggest that nutritional management functions not only as a practical caregiving task, but also as an expression of care, protection and ethical commitment within the household.

Like others, (87, 126) caregivers in the present study were eager to feed their children and pets with the highest quality diet possible. However, this aspiration was tempered by barriers, including financial and time pressures. In the absence of a single “*best*” functional food for optimal health (127) caregivers were often required to make trade-offs between products and product attributes when making food purchasing decisions, a process consistent with discrete choice analysis (128). Consequently, caregivers selected food they considered to provide the highest utility level among the available choices and within the constraints of daily life.

Many caregivers in the present study used visual assessments as an indication of dietary appropriateness for both their children and pets, yet this approach is heavily reliant on extreme and exceptional body condition as a reference point (129). Timely identification of an unhealthy diet, bodyweight, and body condition is crucial for preventing nutrition-related disease and long-term health risks across species (31, 121). However, this process often relies on caregiver recognition, which has been proven inaccurate, in the context of child (129–135) and pet care (136–146).

### 4.2 Key factors influencing caregivers’ choice of food

Caregivers expressed concern for health and responsibility for providing nutritionally adequate diets. However, marked differences emerged between children and pets in the degree of autonomy attributed to the recipient, the influence of trust and expertise, and the emotional and practical dynamics underpinning diet choice.

A myriad of factors have been shown to influence what caregivers choose to feed their children (32, 41, 79, 82, 124, 147–149) and their pets (17, 23, 87, 128, 150–153). Practical constraints, in particular, time, cost, quality and convenience, shaped diet choice for both children and pets. On busy weekdays, convenience was a priority for children’s meals, resulting in caregiver reliance on familiar foods that children readily accepted. Consistent with a global study of 2,400 parents (154), caregivers in the present study aimed to provide healthy meals while also desiring quick and easy meal preparation. Cost pressures also influenced food choice, occasionally limiting access to preferred or perceived healthier food options. For pets, mid-range commercial diets were viewed as a compromise between affordability and perceived quality. Cheaper options were often viewed as inferior, while premium options were considered desirable, yet financially nonviable. Similar to previous research (119, 150) routine and habit further shaped pet food choice, with many caregivers influenced by past experience and the traditions of friends and family.

Although feeding relationships were bidirectional in both child and companion animal contexts, children’s influence stemmed primarily from their perceived agency and developing autonomy, whereas pets’ influence reflected caregivers’ responses to learned food-seeking behaviors and the emotional dynamics of the human–animal relationship. Food preferences and aversions play an important role in caregivers’ dietary decision making with children’s opinions, in particular shaping decisions around main meals (155, 156). Feeding children was often a challenging dynamic, in which caregivers’ balanced nutritional ideals with children’s preferences and developing autonomy, consistent with responsive feeding literature (47, 77, 157). In distinct contrast, pets were positioned as more dependent recipients of care whose dietary preferences were interpreted primarily through the lens of health rather than personal choice. Nevertheless, caregivers described responding to behaviors such as vocalization, and perceived facial expressions, which often elicited treat-giving or additional food. These responses reflected the emotional nature of the human–animal bond and, in some cases, the anthropomorphic interpretation of pets’ behaviors, although typically without the sustained negotiation or accommodation of autonomy described in child feeding (158).

The contrasting interpretation of food refusal illustrates how the perceived status and agency of the recipient shaped caregivers’ responses. Refusal by pets was more readily interpreted as a potential sign of illness, whereas in children, similar behavior was frequently normalized as an expression of autonomy and managed through negotiation. Consequently, caregivers were attentive to their pet’s dietary preferences and fed the same diet long term to avoid health issues, nutritional risks or costs associated with refusal. Conversely, caregivers were more likely to adapt and vary their children’s diet, balancing dietary guidance with personal judgment and children’s preferences. These contrasting dietary choices highlight how assumptions about autonomy influence feeding behaviors across multispecies households. Interestingly, pet caregivers appeared to justify their dietary choices for pets, but not for their children.

Comparable to Fisk et al. (125), findings of the current study suggest that caregivers’ personal beliefs and lifestyles shaped children’s dietary choices, with many describing the transmission of personal values while recognizing that children would ultimately develop independent dietary preferences. In contrast, most caregivers prioritized their pets’ species-specific nutritional needs over personal dietary values. For example, those following plant-based diets for themselves and their children, recognized the inappropriateness of this diet for cats as obligate carnivores.

Similar to Goldthorpe (116), participants were motivated to feed a diet perceived as balanced, nutritious, and conducive to long-term health, however the way nutritional adequacy was operationalized differed. For children, nutrition was actively negotiated and frequently discussed, with caregivers demonstrating awareness of macronutrient balance and the importance of fruit and vegetable intake. In contrast, and comparable to other literature (87, 159–161), most caregivers in the current study favored commercially-produced pet diets perceived as nutritionally complete and trustworthy. In these instances, brand loyalty was apparent, with many selecting the same flavor and type of diet.

In the present study, a minority fed home-prepared pet food, commonly using ingredients similar to their own diets, despite limited consideration of nutritional adequacy or species-specific requirements. Like Kittitunyapong et al. (162), these findings suggest that inappropriate feeding reflects not only knowledge gaps but also broader influences. Comparable to the findings of others (119, 121, 163), few caregivers reported receiving nutritional counseling from medical and veterinary healthcare professionals but, when accessed, this appeared to support long-term diet consistency, particularly for dependents with health-related conditions.

### 4.3 Key factors influencing caregivers’ feeding practice

The current study highlighted the influence of habit, routine and caregiver responsibility in the feeding practice of children and pets, reflecting a One Health interplay within shared domestic environments (121, 164). Participants were the primary dietary gatekeepers for both children and companion animals. A particularly clear divergence concerned the factors underpinning feeding practices. Child feeding was constructed as socially and emotionally embedded, shaped by developmental needs, normative guidance, and relational goals, whereas pet feeding was more heavily influenced by habit, convenience, and perceived nutritional adequacy.

Consistent with Goldthorpe (116), personal upbringing, habit and tradition emerged as shared influences shaping feeding practices for both children and pets. Caregivers described how their own upbringing influenced children’s mealtime routines, suggesting intergenerational transmission of feeding practices. Pet feeding was similarly habitual, but routines were rarely questioned or revised unless prompted by a salient trigger, such as veterinary advice or changes in bodyweight. Thus, feeding routines were central to both contexts, but more adaptable for children.

The divergence in shared mealtimes illustrated the relationally contingent nature of feeding. For children, eating was embedded within family routines and socialization, with shared mealtimes valued for family connection, communication, and the modelling of eating behaviors. Table-based eating was prioritized for safety, social engagement, and behavioral regulation, even when children’s attention or preferences challenged these ideals. In contrast, pet feeding was largely asynchronous to the timing of human meals. This suggests that although pets were frequently constructed as family members, their incorporation into family life did not necessarily extend to all practices associated with human kinship. That said, while feeding interactions do not necessarily cause a social bond (165), receiving or providing food is considered a primary means of human expression of care (166) and is linked to social bonding (167).

In the present study, practical considerations, including time, work schedules and household composition, shaped feeding practices. Children’s weekday meals were structured around school and activities, whereas pet feeding was more closely aligned with caregiver availability and convenience. Similar to another recent study (160), most cats and dogs were fed twice daily, with cats more likely to be fed ad libitum than dogs. Portion sizes were often estimated rather than measured, with pet feeding particularly susceptible to inconsistency between caregivers and pets’ behavioral manipulation. Consistent with Kamleh et al. (150), packaging information and assessments of body condition and activity also influenced pet portion sizes.

Emotional motivations shaped feeding in both contexts but manifested differently. For children, caregivers’ responses centered on long-term health, behavior, and developing healthy relationships with food. Food refusal elicited strong emotional responses, including frustration and anger, reflecting the time, patience and emotional labor associated with feeding children. Yet caregivers encouraged intake while maintaining a positive feeding environment, with adequate food consumption judged over one or more days, rather than per meal. In contrast, pet feeding was more strongly associated with guilt, affection, and embarrassment. Treat-giving often served to express affection, reinforce the human-animal bond or manage behavior, while pet weight gain prompted guilt, shame and changes in feeding practice following veterinary advice. These findings highlight the potential value of family-focused nutrition education informed by a One Health perspective, particularly given the acknowledged parallels in obesity across pets and children (9, 168).

### 4.4 Caregivers’ level of attachment to pets compared to children

Emotional bonds can shape perceptions of responsibility, deservingness, and vulnerability, which in turn affect decisions about the quality, type, and allocation of food given to dependents. Consistent with a UK based survey of 2,000 pet caregivers (10) and an international survey of over 19,000 dog and cat caregivers and 1,500 veterinarians (5) pets in the present study were considered family members. Yet, as per the outcomes from previous research (169–171), construction of the pet-parenting role and attachment orientation of caregivers to their pets differed according to the presence of children. Most participants in the current study were already pet caregivers and originally considered them a child substitute, “*our babies before we had our babies*”, yet the presence of young children resulted in a lack of time, energy and attention to afford pets.

The current findings suggest that parenthood reshaped, rather than diminished, attachment to pets as caregiving priorities shifted toward children. This implicit hierarchy of care, was often accompanied by guilt and emotional ambivalence about perceived pet neglect, consistent with others (170, 172). Attachment was also influenced by pets’ perceived independence and their integration into family routines and relationships with children, suggesting that it is dynamic and responsive to caregiving demands (173). The household therefore represents an important One Health context in which caregiver beliefs and practices shape the wellbeing of both humans and companion animals.

### 4.5 Strengths and limitations of the present study

The results of this qualitative research are specific to the setting. Readers should consider the study’s context, methodology, and the authors’ positionality when assessing the applicability of the findings.

A major strength of this study was its direct comparison of the dietary decision making and feeding management of caregivers of both pets and children in same household, something which, to the authors’ knowledge, has not been examined previously. Findings can be understood through a One Health lens, recognizing the interconnected nature of human and animal health within a shared household environment. They further demonstrate that caregiving beliefs and feeding philosophies transfer across contexts in complex, sometimes contradictory, ways. Recognizing commonalities and distinctions in child and pet feeding practices offers valuable opportunities for more integrated, family-focused nutrition education and guidance. By addressing misconceptions, contextual constraints, and emotional drivers, future interventions can, potentially at least, better support evidence-based feeding decisions and improve health outcomes for both human and animal dependents.

A convenience strategy was used to recruit participants. The study included a relatively small, purposively selected sample of UK-based caregivers of children aged up to ten years and dogs and/or cats, enabling an in-depth exploration of participants’ experiences within this specific context. As such, the findings reflect a Western perspective on caregiving and companion animal ownership. Further research is needed to explore whether similar feeding decision-making processes are evident in other cultural and geographic contexts.

Whilst efforts were made to select participants according to varied characteristics (age, gender, educational attainment, diet preference, etc.), participants may have had different views about diet choice and feeding practice to those who chose not to participate. The sample comprised a slightly higher proportion of female than male participants, however this reflected the gender distribution of those who expressed interest in participating. The findings were based on participants’ retrospective accounts and personal interpretations of their experiences. As such, these accounts may have been shaped by participants’ current perspectives, memory, and willingness to disclose information. Whilst findings could be transferable, they cannot be generalized to the wider population of child and pet caregivers and so future replication of the study with a larger and more diverse population is recommended.

Examination of socioeconomic status (SES) was not a key focus of the current study, however socioeconomic inequalities is an established factor influencing the healthiness of food choices (174, 175). Food and nutrition-related beliefs are also likely shaped by people’s experiences, associated with their demographic status (176). Educational attainment is an important sociodemographic variable that influences dietary behavior and choice (177–179). As part of the recruitment process for the current study, participants were asked for details of their highest educational attainment. The sample had a high level of educational attainment, with most participants holding university degrees. As educational attainment is commonly used as a proxy for socioeconomic position, this suggests the sample may have been relatively socioeconomically advantaged, therefore findings may not fully reflect the experiences of more diverse or disadvantaged populations.

### 4.6 Future implications

Both pets and children rely heavily on caregivers for nutritional intake, highlighting the importance of targeted nutrition education and guidance. By comparing caregivers’ feeding practices and diet choices across these contexts, this study provides valuable insight into how caregiving beliefs, feeding philosophies and nutritional attitudes may be transferred across contexts, while also distinguishing important divergences in how these are enacted. Nutrition was considered a central marker of responsible care, but caregivers described tensions between nutritional ideals and practical, financial and emotional realities. These findings have several implications. First, nutrition education should extend beyond the provision of information to address the beliefs, expectations and practical constraints that influence feeding decisions. Second, education and guidance should acknowledge the different relational and decision-making dynamics involved in feeding children and pets. In particular, the relative stability of pet feeding in the current study highlights a need for veterinary professionals to proactively discuss nutrition with caregivers, rather than addressing it only when health concerns arise. Conversely, the greater proactivity observed in child feeding highlights the importance for medical professionals to support caregivers in translating nutritional aspirations into realistic and sustainable practices. Across both contexts, identifying shared misconceptions and decision-making patterns may help to inform more targeted, evidence-based nutrition communication, while maintaining clear distinctions between species-specific nutritional requirements. Future interventions could therefore benefit from a more context-sensitive approach that recognizes both the common influences on caregiver decision-making and the distinct nutritional, developmental and health needs of children and companion animals. Further comparative research into caregivers’ perceptions of feeding management across different child developmental stages and companion animal life stages would be valuable.

## Data Availability

The data files which underpin this research have been deposited in the University of Glasgow institutional data repository, 10.5525/gla.researchdata.2166 for long-term safekeeping. The data files are not available for reuse due to ethical constraints. Further inquiries can be directed to the corresponding author/s.
