# Supplementary material for: A comparative study of diet choice and feeding management among UK pet and child caregivers
Source: Front Vet Sci. 2026 Oct 12;13:1883885. doi: 10.3389/fvets.2026.1883885 (PMC13701101; doi:10.3389/fvets.2026.1883885)
Supplement: Supplementary file 1 [file Supplementary_file_1.pdf]

Supplementary Material

1 Supplementary File 1: The self-developed online screening questionnaire.

1. Please identify your age in years
    - 18-24
    - 25-35
    - 36-45
    - 46-55
    - 56-65
    - Over 65

2. Which of the following best describes the area you live in?
    - Urban (densely populated inner town or city)
    - Suburban (less densely populated outskirts of a town or city)
    - Rural (based in the countryside)

3. What is your highest level of education?
    - Up to completion of secondary education
    - Post secondary education
    - Undergraduate or equivalent level (e.g. Bachelor's degree)
    - Postgraduate or equivalent level (e.g. Master's / Doctoral degree)

4. Are you employed or involved in the veterinary healthcare field or pet animal industry?

- No
- Yes
    - veterinarian or veterinary nurse
    - student of veterinary medicine, veterinary science or veterinary nursing
    - breeder
    - within an animal service (behaviourist/trainer, groomer, farrier, animal acupuncturist, kennel staff, veterinary receptionist, etc.)
    - within an animal industry (pet food, pet toys, pet products)
    - Other (please state) ___________________

5. Are you employed or involved in the human healthcare or diet and fitness industry?

- No
- Yes
    - healthcare professional – Doctor (generalist or specialist practitioner), Nurse, Midwife, Dentist, Pharmacist
    - student healthcare professional (as listed above)
    - within an allied healthcare service (e.g. physiotherapist, psychologist, paramedic, radiographer, dental hygienist/assistant)
    - within the diet and fitness industry (e.g. dietician/nutritionist, personal trainer/fitness instructor, health coach, health/diet-related sales representative).
    - Other (please state) ___________________

6. What is your personal dietary preference?

- Omnivore (meat and plant-based diet)
- Pescatarian (containing fish, but no other meats)
- Vegetarian (avoiding all meat but including eggs, dairy and/or honey)
- Vegan (free from all animal products)

- ○ Other (please specify)

7. Do you follow an alternative diet? (Tick all that apply)
   - ☐ Gluten-free or another specific food allergy or intolerance (e.g. lactose-free)
   - ☐ Intermittent fasting
   - ☐ Ketogenic diet (low carbohydrate, high fat)
   - ☐ Low carbohydrate diet
   - ☐ Low fat diet
   - ☐ Mediterranean diet (rich in vegetables, fish, fruit, grains, olive oil, and nuts, moderate dairy and minimal red meat)
   - ☐ Paleolithic (paleo) diet (avoiding all processed foods)
   - ☐ Other (please state) ___________________
   - ☐ N/A - I don't follow any alternative diet

8. As a parent or guardian, how many children aged 0-10 years do you have responsibility for feeding?
   - ○ 1
   - ○ 2
   - ○ 3
   - ○ 4
   - ○ 5
   - ○ More than 5

9. What dietary preference do you adopt for the child/children?
   - ○ Omnivore (meat and plant-based diet)
   - ○ Pescatarian (containing fish, but no other meats)
   - ○ Vegetarian (avoiding all meat but including eggs, dairy and/or honey)

- o Vegan (free from all animal products)
- o Other (please specify)

10. Do you feed the child/children any of the following alternative diets? (Tick all that apply)

- ☐ Gluten-free or another specific food allergy or intolerance (e.g. lactose-free)
- ☐ Intermittent fasting
- ☐ Ketogenic diet (low carbohydrate, high fat)
- ☐ Low carbohydrate diet
- ☐ Low fat diet
- ☐ Mediterranean diet (rich in vegetables, fish, fruit, grains, olive oil, and nuts, moderate dairy and minimal red meat)
- ☐ Paleolithic (paleo) diet (avoiding all processed foods)
- ☐ Other (please state) ____________________
- ☐ N/A - I don’t feed the child/children any alternative diet

11. How many dogs and/or cats do you have responsibility for feeding?

Dogs ____

Cats ____

12. What dietary preference do you adopt for each of your pet(s)?

- o Meat-based
- o Omnivore (meat and plant-based diet)
- o Vegetarian (avoiding all meat but including eggs, dairy and/or honey)
- o Vegan (free from all animal products)
- o Other (please specify)

13. What type of diet do you feed to your pet(s)? (Tick all that apply).

- ☐ Commercial cooked diet (dry/kibble or wet/canned/tinned/sachet)
- ☐ Home-prepared cooked diet
- ☐ Commercial raw meat diet
- ☐ Home-prepared raw meat diet
- ☐ Combination of commercial and homemade diet (cooked)
- ☐ Combination of commercial and homemade diet (raw meat)
- ☐ Freeze-dried or rehydrated commercial pet foods
- ☐ Grain-free diet
- ☐ Human-grade fresh or frozen seafood
- ☐ Novel protein diet (e.g., insect based)
- ☐ Prescription/therapeutic diet (e.g. food allergy, liver-care, kidney-care etc.)
- ☐ Raw bones
- ☐ Weight loss diet
- ☐ Other (please specify) ___________

2 Supplementary File 2: The semi-structured interview script.

Thank you for giving up your time to talk to me today. I'd like to start the interview by asking you about the feeding management of your dependents across a typical week. Please could you describe their meals and food intake to me?

Dietary-related decisions are often formulated and affected, or influenced, by a range of factors, such as social, educational, cultural and marketing factors.

1. When feeding your child/children, what key factors influence your:

    a. choice of diet (e.g. type of foods) and

    b. feeding behaviour/practice (e.g. strategies that inform when, what, and how much food is eaten)?

        - Can you expand to explain why?
        - Any positive or negative influences?
        - What about any personal values and/or beliefs?

2. When feeding your pet(s), what key factors influence your:

    a. choice of diet (e.g. type of foods) and

    b. feeding behaviour/practice (e.g. strategies that inform when, what, and how much food is eaten)?

        - Can you expand to explain why?
        - Any positive or negative influences?
        - What about any personal values and/or beliefs?

3. How important do you believe nutrition is for good health and wellbeing?

4. What is your understanding of minimally processed foods?

    - Are you able to provide an example of a minimally processed food product for a) your pet(s) and b) your child/children?

- In what situations would you feed or avoid feeding these types of food?

5. What **benefits** do you believe to be associated with feeding (predominantly) fresh, unprocessed, or minimally processed foods?

6. What **disadvantages** do you believe to be associated with feeding (predominantly) fresh, unprocessed or minimally processed foods?

7. What is your understanding of ultra-processed foods?

   - Are you able to provide an example of an ultra-processed food product for a) your pet(s) and b) your child/children?
   - In what situations would you feed or avoid feeding these types of food?

8. What **benefits** do you believe to be associated with feeding (predominantly) a commercially-produced, ultra-processed diet?

9. What **disadvantages** do you believe to be associated with feeding (predominantly) a commercially-produced, ultra-processed diet?

10. In comparison to your child/children, how would you describe your level of attachment to your pet(s)?
